# Expression of Endoplasmic Reticulum Chaperones in Cardiac Develop-ment

**DOI:** 10.2174/1874192400802010031

**Published:** 2008-05-21

**Authors:** Sylvia Papp, Xiaochu Zhang, Eva Szabo, Marek Michalak, Michal Opas

**Affiliations:** 1 Department of Laboratory Medicine and Pathobiology, University of Toronto, Toronto, Ontario, Canada; 2 Department of Biochemistry, University of Alberta, Edmonton, Alberta, Canada

**Keywords:** Heart, endoplasmic reticulum stress, chaperones, unfolded protein response, embryonic development, apoptosis

## Abstract

To determine if cardiogenesis causes endoplasmic reticulum stress, we examined chaperone expression. Many cardiac pathologies cause activation of the fetal gene program, and we asked the reverse: could activation of the fetal gene program during development induce endoplasmic reticulum stress/chaperones?

We found stress related chaperones were more abundant in embryonic compared to adult hearts, indicating endoplasmic reticulum stress during normal cardiac development. To determine the degree of stress, we investigated endoplasmic reticulum stress pathways during cardiogenesis. We detected higher levels of ATF6α, caspase 7 and 12 in adult hearts. Thus, during embryonic development, there is large protein synthetic load but there is no endoplasmic reticulum stress. In adult hearts, chaperones are less abundant but there are increased levels of ATF6α and ER stress-activated caspases. Thus, protein synthesis during embryonic development does not seem to be as intense a stress as is required for apoptosis that is found during postnatal remodelling.

## INTRODUCTION

In the endoplasmic reticulum (ER) lumen, the near neutral pH, Ca^2+^ homeostasis, and the existence of the protein folding machinery as well as the quality control system are all necessary for proteins to be correctly synthesized and folded [1]. Any perturbations to ER homeostasis will induce ER stress, which is characterized by the accumulation of misfolded or unfolded proteins within the ER lumen, which may induce the unfolded protein response (UPR) [1-3]. The UPR is a transcriptional and translational signaling network, initiated by the accumulation of misfolded and unfolded proteins in the ER lumen [4]. The UPR has several key players, including activating transcription factor 6 (ATF6), which is an ER membrane-anchored transcription factor. ATF6 is transported from the ER to the Golgi upon ER stress and is cleaved during this stress response [2, 5]. The movement of ATF6 between the ER and Golgi is controlled by ER chaperone Grp78, also known as BiP, which belongs to the glucose regulated protein family of ER stress proteins. Another member of the UPR is IRE1, an ER-resident protein kinase and endonuclease, which ultimately allows ER chaperone gene promoters to be activated [6]. Finally, PKR-like ER kinase (PERK) phosphorylates eukaryotic initiation factor 2α (eIF2α) during ER stress, thereby attenuating mRNA translation and reducing the load for the ER folding chaperones [7]. In summary, the UPR is activated by the dissociation of Grp78 from the ER stress transducers: PERK, IRE1 and ATF6  [8, 9].

The endoplasmic reticulum (ER)- resident chaperone, calreticulin, has been found to play an important role in embryonic heart development, as calreticulin deficiency is embryonic lethal [10, 11]. Calreticulin-null mice die *in utero* due to severe cardiac malformations, such as extreme ventricular thinning and a deepening of the intertrabecular recesses. It is evident, then, that embryonic chaperoning by calreticulin is imperative for proper cardiac development. One common function of calreticulin and other ER resident chaperones is to assist in the folding of newly synthesized proteins within the ER lumen. Such chaperoning is especially crucial early on in development, when the synthetic load of the embryo is extremely high. For example, during mouse heart development, the expression of two ER resident stress chaperones, glucose regulated protein 94 (Grp94) [12] and glucose regulated protein 78 (Grp78) [13], was found to be significantly higher at early gestational days (E9.5-13.5) than at later days (E19.5).

In this study, we wished to first examine whether or not stress related ER-resident chaperone expression changed during development. In addition, we wanted to know if ER stress existed during embryonic development. Calreticulin is a chaperone that is regulated during cardiac development [14] and its absence causes ER stress and UPR [15], which may lead to apoptosis [16]. In recent years, accumulating data has suggested that there is a relationship between ER stress and apoptosis. It thus is necessary to determine whether ER stress is a mechanism that induces apoptosis during embryonic development. Two ER stress activated caspases, caspase-7 and caspase-12 were examined here to test this hypothesis. ER stress (and UPR) [17-21] as well as apoptosis itself [22], have been associated with cardiomyopathies [17-21]. Tremendous cellular remodelling occurs during cardiac pathologies, and cardiac remodelling is associated with ER stress and apoptosis. Virtually nothing is currently known about ER stress proteins and cardiac remodelling in normal development. Thus, the aim of this study was to determine the degree, if any, of ER stress during normal cardiac development, by examining the expression patterns of ER-resident, stress related chaperones as well as by examining the expression of UPR and apoptotic markers.

## MATERIALS AND METHODOLOGY

### Animals

Friend leukemia virus B-sensitive mice (FVB) mice were used. Timed pregnancies were performed by randomly breeding FVB mice and checking vaginal plug formation each morning. Observed vaginal plugs were designated as gestational day 0 (denoted as E0). At an experimentally specified gestational day (day 13 to day 18, i.e., E13 to E18), pregnant FVB mice were sacrificed by cervical dislocation, embryos removed and hearts were dissected out. The tissues were then used for Western blot analysis. Minimum 15 embryonic hearts were used for atrial preparations and 10 for ventricular. As it was impossible to separate atria from ventricles at E13, minimum 10 whole embryonic hearts were used for each preparation. For adult atrial and ventricular preparations, non-pregnant animals approximately 6 months old were used.

### Cell Culture

To provide a positive control for experiments with western blotting of ATF6α, mouse embryonic fibroblasts were grown on high-glucose Dulbecco’s Modified Eagle’s Medium supplemented with 10% fetal bovine serum and 2 mM L-glutamine. The cells were grown to 80% confluence in tissue culture dishes followed by overnight treatment with thapsigargin (500 nM). Cells were collected the next day and were subjected to Western blot analysis.

### Western Blotting

Cells and tissues were homogenized in lysis buffer (1 M Tris-Cl PH 8.0, 1.2 M NaCl, 0.5% v/v Nonidet P-40) with 10 μl/ml of protease inhibitor cocktail freshly added (Sigma) for 15 minutes on ice, followed by centrifugation at 14000 X g for 15 minutes at 4°C. Supernatants were then measured for protein content according to the manufacturer’s instructions (Bio-Rad). Samples were boiled for 5 minutes in reducing sample buffer with 2-mercaptoethanol (5 μl/100ml) and then subjected to 10% SDS-PAGE. The separated sample proteins were transferred to nitrocellulose membranes, which were then blocked in 5% milk in PBS with 0.01% Tween-20 (PBST) overnight at 4°C. After blocking, membranes were incubated in diluted primary antibodies for 1 hour at room temperature, followed by washing in PBST for 15 minutes (3 times). Membranes were then incubated in diluted (1:10000) secondary horseradish peroxidase (HRP) conjugated antibodies for another 1 hour, followed by washing in PBST for 15 minutes (3 times). ECL Plus Western blotting detection reagents (Amersham Biosciences) and Kodak x-ray film were utilized to visualize the protein bands. Primary antibodies diluted in PBST were as follows: goat anti-calreticulin, 1:300; rabbit anti-Grp94 1:1000; rabbit anti-PDI 1: 1000; rabbit anti-ERp57 1:1000; rabbit anti-ATF6α polyclonal antibody 1: 200 (Santa Cruz); rabbit anti-calnexin polyclonal antibody (spa-860) 1: 2000 (Stressgen Biotechnologies); rabbit anti-caspase-12 polyclonal antibody 1:100 (Cell Signaling Technology); rabbit anti-caspase-7 polyclonal antibody 1:1000 (Cell signaling Technology); rabbit anti-Grp78 polyclonal antibody 1:2000 (Stressgen Biotechnologies); rabbit anti-glyceraldehyde-3-phosphate dehydrogenase (GAPDH) 1:2000 (Labfrontier, Korea). The protein bands in each blot were normalized by using GAPDH antibody and relative protein levels were quantified using Image J software.

## RESULTS AND DISCUSSION

### Differential Expression of Calreticulin and Calnexin During Heart Development

**Fig. (1):**
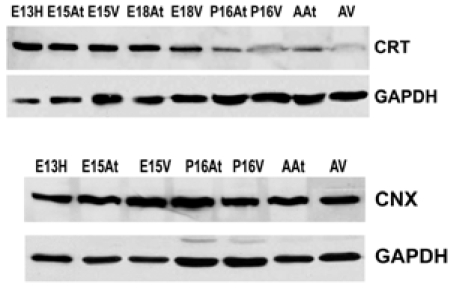
**Calreticulin and calnexin expression during mouse heart devel-opment. A:** Western blotting shows that calreticulin (CRT) is more abundant in embryonic hearts compared to adult hearts. Its expression is dramatically down-regulated postnatally. In contrast, calnexin in (CNX) expression does not change during embryonic development or into adulthood. GAPDH was used as a loading control.E – embryonic day; P – postnatal day; A – adult; H – heart; At – atria; V – ventricles

There are several families of ER chaperones, which work in co-ordinated pairs and/or groups. The calreticulin/calnexin cycle of chaperoning has been well established [23-25]. We first investigated the expression pattern of this pair of chaperones during heart development. We have previously shown that calnexin is not induced by ER stress, but that calreticulin is indeed inducible by ER stress [26]. Fig. (**1A**) shows that calreticulin expression was higher in embryonic heart tissues compared to their adult counterparts. In particular, calreticulin expression diminished dramatically in ventricular tissue postnatally, and remained low into adulthood (Fig. **1**, Fig. **4**). Earlier studies have demonstrated a role for ER resident chaperones in embryonic development using knockout mice for specific chaperones [10, 27, 28]. Two chaperones, such as calreticulin and Grp94, were found to be critical in embryonic development, as their deficiency induced embryonic death. When calreticulin was ablated, calreticulin-deficient mouse embryos died at gestational day 14.5 as a result of faulty cardiac organogenesis. The cardiac structural defects included very thin ventricular walls with deep intertrabecular recesses [10, 11]. In line with this gene knockout study, our current data showed higher expression of calreticulin in both mouse embryonic atrial and ventricular tissue than in the adult. Calreticulin expression began to subside 17 days postnatally in both atria and ventricles, and in adult ventricles, calreticulin was barely detectable.

Calnexin expression, on the other hand, remained largely unchanged during cardiac development, as well as into adulthood (Fig. **1B**). Calnexin deficiency is not embryonic lethal. Calnexin-null mice are viable but exhibit ataxia [27] (and unpublished data from Dr. Michalak). Our data showed that calnexin expression did not change during mouse heart development [26], which may indicate different roles for calnexin and calreticulin during embryonic development. This is not surprising, as calreticulin and calnexin, which often work in tandem as chaperones, also have unique substrates [23, 29]. In addition, calreticulin is a soluble ER lumenal protein, whereas calnexin is a type I ER membrane protein, which may also lead to their differences in function [15]. We have previously demonstrated that ER protein distribution contributes to the heterogeneous function of different ER domains [30].

### Grp94 and Grp78 Expression in Embryonic Heart Development

**Fig. (2):**
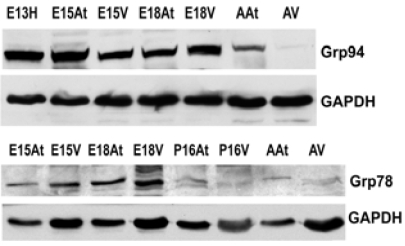
**Expression of Grp94 and Grp78 during mouse heart development.** . The expression of both Grp94 and Grp78 is higher in embryonic hearts than adult hearts. This reflects protein levels in both the atria and the ventricles. GAPDH was used as a loading control.E – embryonic day; P – postnatal day; A – adult; H – heart; At – atria; V – ventricles

**Fig. (4):**
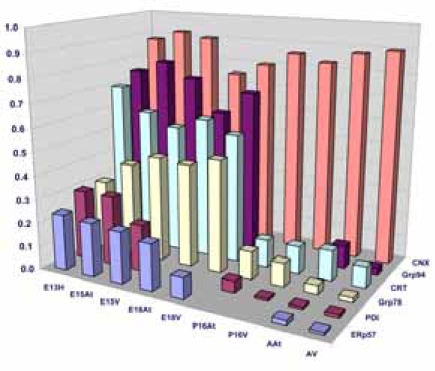
**Quantification of ER-resident chaperone levels during mouse heart development.** .Quantification of Western blots of ER-resident chaperones. The bar graphs denote an average of minimum 4 experiments. The chaperone protein levels are expressed as a ratio of GAPDH as described in Materials and Methods

An important class of ER chaperones are the glucose-regulated proteins (Grps), which belong to the heat shock family of stress proteins. Grp78, also known as BiP, and Grp94 function as molecular chaperones and can bind to either misfolded proteins or unassembled polypeptides. They are induced in response to stress, but are then post-transcriptionally modified into inactive forms upon removal of the stress [31]. In order to detect the expression of Grp94 and Grp78 during embryonic development, Western blot analyses were performed on mouse heart tissues at embryonic and adult stages. Both Grp94 and Grp78 exhibited similar expression levels as that observed for calreticulin during mouse heart development (Fig. **2A**, **B**, Fig. **4**). In all tissues examined, Grp94 and Grp78 expression was higher in embryonic *versus* the adult tissues. Barnes and Smoak [12, 13], showed that Grp94 and Grp78 levels in the heart were highest when the myocardial cells were proliferating rapidly, indicating a high demand for the synthesis of these two proteins at certain stages of heart development. Our present data shows that Grp78 and Grp94 expression is high in both atria and ventricles until gestational day 18. At postnatal day 15, Grp78 expression level in the atria and ventricles is very low, and not detectable in the adult, indicating that there may also be a peak in the regulation of Grp78 during mammalian heart development. These data indicate that Grp78 and Grp94 play an important role during cardiomyocyte differentiation and heart development.

### PDI and ERp57 Expression During Heart Development

Another class of chaperones we examined were the protein disulfide isomerase (PDI) family, which assist in disulfide bond formation. We specifically examined PDI and ER protein 57 (ERp57), which is an ortholog of PDI that acts on glycosylated proteins [32]. To examine the expression of PDI and ERp57 during mouse embryo development, we performed Western blot analyses of PDI and ERp57 in mouse embryonic and adult heart tissues. Fig. (**3A**, **B**) and Fig. (**4**)show that the higher expression levels of ERp57 and PDI were during embryonic development, while these proteins are barely detectable in adult tissues. This expression pattern is similar to that of calreticulin, Grp94 and Grp78.

In summary, three groups of ER resident stress-related chaperones have been found to have a similar pattern of expression during mouse embryonic development, and are all greatly downregulated in adult tissues. We thus conclude that there is a form of ER stress during normal cardiac development. We next sought to investigate the degree of this ER stress in embryos compared to the degree of stress in adulthood, in order to understand the mechanism behind this upregulation in the embryonic stages. Is it a large synthetic load to which these chaperones are responding or is embryonic development also accompanied by a form of ER stress?

### Detection of UPR factors in Heart Development

In order to investigate whether the UPR, an ER stress initiated signaling network, is also detectable in embryonic development, we examined several factors in each UPR pathway.

**Fig. (5):**
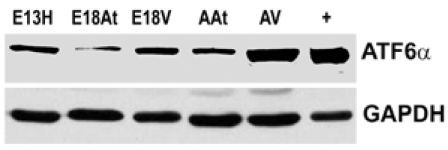
**Detection of ATF6α in mouse heart tissues.** . Western blotting of ATF6α reveals that the protein is more abundant in adult heart tissue, especially adult ventricular tissue, compared to embryonic heart tissues. Lane “+” refers to the positive control for ATF6α which was an extract from the thapsigargin treated mouse embryo fibroblasts as described in Materials in Methods.GAPDH was used as a loading control.E – embryonic day; A – adult; H – heart; At – atria; V – ventricles

We could not detect eIF-2α protein,phospho-eIF-2α protein or cleaved XBP-1 mRNA (a cleavage product of IRE1) either during embryonic heart development or during adulthood (data not shown). We also performed Western blot analysis of ATF6α expression in mouse embryonic heart tissues. The expression of ATF6α remained unchanged during embryonic heart development and was slightly higher in adult tissues, especially in the adult ventricles (Fig. **5**). It seems then, that there is not a great degree of ER stress within the developing mouse heart. We could not detect UPR activation in developing mouse hearts, but there seemed to be some degree of ATF6 induction in the adult heart. It can only be speculated on that such an induction may be due to cardiac remodelling. Further examinations need to be undertaken to determine the degree of such possible remodelling and to determine if it is normal or pathologic.

### ER-Stress Activated Caspases During Heart Development

To further determine the degree of ER stress in developing and adult mouse hearts, Western blot analysis was performed for caspase-7 and caspase-12, which have been shown to be activated during ER stress [33, 34]. The antibodies used here were able to detect both pro-caspases and cleaved caspases 7 and 12. Fig. (**6**) shows that both pro-caspases and cleaved caspases were much more abundant in embryonic ventricles than in atria. More importantly, these caspases were more abundant in adult hearts compared to embryonic hearts, suggesting a greater degree of apoptosis in adult hearts, which may be related to cardiac remodelling.

In summary, in terms of markers of UPR activation and markers of apoptosis, there seems to be a greater degree of ER stress in adult hearts than embryonic hearts. This is contrary to the high degree of ER stress found in embryonic eye and brain [26]. In the embryonic heart, although ER-resident stress-related chaperones are more abundant than in the adult, ATF6α is decreased and eIF2α and XBP-1, components of the UPR, are not detectable. Thus, during embryonic heart development, the driving force for the extensive upregulation of ER stress chaperones seems to be the great synthetic load and not ER stress. On the other hand, in adult hearts, ER-resident stress-related chaperones are less abundant but there are increased levels of ATF6α and the ER stress-related caspases 7 and 12. It seems, then, that protein synthesis (synthetic load) during embryonic development is not as intense a stress as is required for apoptosis that is found during postnatal remodelling. Thus, during embryonic heart development, ER stress-related chaperones are activated but the UPR is not activated and neither is apoptosis induced to the levels found in adult hearts. The ER chaperones are an important part of a “quality” control” machinery of the ER [35], and may participate in UPR and ER stress, which may in turn lead to apoptosis [16]. ER stress (and UPR) [17-21] as well as apoptosis itself [22], have been associated with cardiomyopathies. Postnatal remodelling of the heart normally occurs but it may also be related to a variety of cardiac pathologies, such as cardiac hypertrophy and dilated cardiomyopathies.

## CONCLUSIONS

In the embryo, although endoplasmic reticulum-resident chaperones are more abundant than in the adult, ATF6α is decreased and eIF2α and XBP-1, components of the unfolded protein response, are not detectable. Thus, during embryonic development, there is large synthetic load but maybe there is no endoplasmic reticulum stress. On the other hand, in adult hearts, endoplasmic reticulum-resident chaperones are less abundant but there are increased levels of ATF6α and caspases. It seems, then, that protein synthesis during embryonic development is not as intense a stress as is required for apoptosis that is found during postnatal remodelling, be it normal or pathologic.

## Figures and Tables

**Fig. (3):**
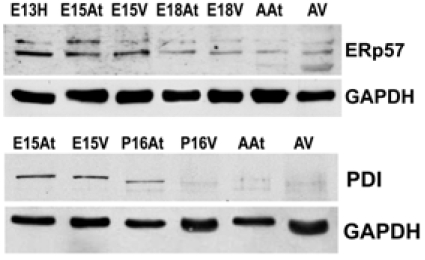
**Expression of ERp57 and PDI during mouse heart develop-ment.** .The expression of both Erp57 and PDI is higher in embryonic hearts than adult hearts. This reflects protein levels in both the atria and the ventricles. GAPDH was used as a loading control.E – embryonic day; P – postnatal day; A – adult; H – heart; At – atria; V – ventricles.

**Fig. (6):**
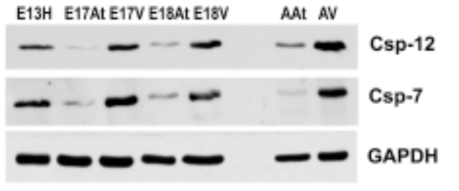
**Western blot analysis of caspase-7 and caspase-12 during heart development. ** .Both caspases are more abundant in embryonic ventricles compared to embryonic atria. Importantly, these caspases are more abundant in adult heart tissues, especially the ventricles, compared to em-bryonic heart tissues. GAPDH was used as a loading control.E – embryonic day; H – heart; At – atria; V – ventricles
